# Adult-onset vanishing white matter disease caused by the EIF2B5 c.185A>T (p.Asp62Val) variant

**DOI:** 10.3389/fgene.2026.1739328

**Published:** 2026-02-02

**Authors:** Jie Zhou, Chunbo Ji, Siqing Ma, Jianying Zhu, Ping Yang

**Affiliations:** 1 Clinical College of Ningxia Medical University, Yinchuan, Ningxia, China; 2 Department of Neurology, General Hospital of Ningxia Medical University, Yinchuan, China; 3 Department of Neurophysiology,General Hospital of Ningxia Medical University, Yinchuan, China

**Keywords:** adult-onset, EIF2B5, genotype–phenotype correlation, homozygous variant, vanishing white matter disease

## Abstract

**Background:**

Vanishing white matter disease (VWMD; OMIM 603896), also known as childhood ataxia with central nervous system hypomyelination (CACH), is a rare autosomal recessive leukodystrophy caused by pathogenic variants in the EIF2B gene family (EIF2B1–EIF2B5). Clinical manifestations are highly heterogeneous, with onset ranging from fetal life to adulthood; adult-onset cases remain relatively rare and often present with atypical symptoms. Brain magnetic resonance imaging (MRI) and genetic testing are pivotal for diagnosis.

**Case Presentation:**

We report a 32-year-old Chinese female with adult-onset VWMD characterized by intermittent headaches, progressive cognitive decline, menstrual irregularities, and hearing loss. Cranial MRI with diffusion-weighted imaging (DWI) revealed symmetrical periventricular and centrum semiovale white matter abnormalities. Whole-exome sequencing (WES) identified a homozygous missense variant in the EIF2B5 gene, formatted per Human Genome Variation Society (HGVS) guidelines as NM_001414.4:c.185A>T (p.Asp62Val). This variant was previously documented exclusively in a pediatric patient, representing the first report in an adult.

**Conclusion:**

Our case expands the phenotypic and age-related spectrum of EIF2B5-associated VWMD, highlighting that the c.185A>T variant is capable of manifesting in adulthood with non-classical features (e.g., headache as the initial symptom). Prior studies have confirmed that this variant impairs EIF2B complex function, which reinforces its pathogenic role in disrupting the integrated stress response (ISR) and maintaining white matter homeostasis. A literature review of 99 genetically confirmed adult-onset VWMD cases further underscores genotype–phenotype correlations: EIF2B5 is the most frequently mutated subunit in adult patients, with cerebellar ataxia, cognitive decline, and psychiatric symptoms as the predominant initial manifestations. Female patients often present with premature ovarian failure, a key diagnostic hallmark. Early genetic testing is crucial for definitive diagnosis, prenatal counseling, and symptomatic management. Notably, this study has limitations, including the lack of investigation into gene-gene interactions—factors that may modulate disease severity and phenotypic variability—and the unavailability of parental genetic data to fully validate zygosity.

## 1 Introduction

Vanishing white matter disease (VWMD) is a progressive leukodystrophy caused by biallelic pathogenic variants in EIF2B1–EIF2B5, which encode the five subunits (α, β, γ, δ, ε) of the eukaryotic translation initiation factor 2B (eIF2B) complex (Leegwater et al., 2001). This complex regulates mRNA translation initiation and protein synthesis—processes essential for cellular homeostasis (Oba et al., 2025). In VWMD, physical or psychological stress triggers the integrated stress response (ISR), reducing EIF2Bε (encoded by EIF2B5) expression and activity. This impairment perpetuates ISR activation, forming a deleterious feedforward loop that disrupts the unfolded protein response and dysregulates cell proliferation, survival, and apoptosis (Keefe et al., 2020).

Neuropathologically, VWMD is defined by cavitating orthochromatic leukodystrophy, diffuse white matter (WM) vacuolation, and increased oligodendrocyte density with foamy cytoplasm—features that distinguish it from other leukodystrophies (Rodriguez et al., 1999). Radiologically, brain MRI reveals diffuse, symmetrical WM abnormalities that progressively acquire cerebrospinal fluid (CSF)-like signal intensity due to liquefactive necrosis, the hallmark “vanishing” phenomenon (van der Knaap et al., 1997).

VWMD exhibits broad age-related phenotypic heterogeneity, with subtypes classified by onset: congenital, infantile (≤1 year), early childhood (two to four years), juvenile (5–15 years), and adult (>15 years) (Wei et al., 2019). Childhood-onset disease is most prevalent, while adult-onset cases account for a minority of reports (Wei et al., 2019). Age of onset is the sole independent predictor of severity—earlier onset correlates with more aggressive progression (Deng et al., 2021). Adult-onset VWMD often presents with atypical symptoms, such as headaches, cognitive impairment, or psychiatric manifestations, complicating diagnosis (Damásio, 2012; Ho et al., 2020). A subset of female patients develops premature ovarian failure (POF), a syndrome termed ovarian leukodystrophy (OLD), most commonly associated with EIF2B mutations (Lynch et al., 2016).

Here, we report an adult female with VWMD harboring the EIF2B5 c.185A>T (p.Asp62Val) variant—previously described only in a child (Leng et al., 2011) —and conduct a systematic literature review to synthesize genotype–phenotype correlations in adult-onset VWMD. This case expands the variant’s age-related spectrum and highlights critical diagnostic considerations for clinicians.

## 2 Literature search methodology

A systematic literature search was conducted in PubMed (as of 11 July 2024) using the keywords: “vanishing white matter disease”, “adult-onset vanishing white matter disease”, “EIF2B”, “leukodystrophy”, and “ovarian leukodystrophy”. Inclusion criteria were: (1) adult-onset VWMD (onset >15 years); (2) definitive genetic confirmation (pathogenic/likely pathogenic variants in EIF2B1–EIF2B5); (3) full-text availability in English or Chinese. Exclusion criteria were: (1) juvenile-onset disease (≤15 years); (2) unconfirmed genetic diagnosis; (3) case reports with insufficient clinical/imaging data. A total of 99 eligible cases were retrieved, forming the basis of our genotype–phenotype analysis.

## 3 Case presentation

### 3.1 Clinical history

A 32-year-old Chinese female presented to our hospital in September 2021 with frequent, severe headaches, binaural tinnitus, and hearing loss. She first developed intermittent headaches at 20 years of age, localized to the frontal, temporal, parietal, and occipital regions. Headaches were characterized by dull distension, irregular frequency, and precipitation by emotional agitation or fatigue. Over time, headaches persisted and increased in frequency, accompanied by progressive memory loss. In 2019, she underwent cranial MRI with DWI at a hospital in Xi’an, which revealed symmetrical patchy long T1 and long T2 signals in the bilateral periventricular and centrum semiovale WM. She was prescribed long-term oral pregabalin capsules, citicoline sodium tablets, and idebenone tablets, but symptomatic relief was suboptimal.

The patient had no significant medical history. She married at 20 years of age and remained childless; her menstrual cycle had recently prolonged. She denied consanguinity between parents or family history of genetic diseases.

### 3.2 Physical examination

Cognitive function: Montreal Cognitive Assessment (MoCA) score = 19/30; Mini-Mental State Examination (MMSE) score = 21/30 (consistent with mild cognitive impairment).

Neurological examination: Cranial nerve findings were unremarkable. Limb muscle strength was Grade 5, with normal tone and coordination. Deep and superficial sensory examinations were unremarkable. Extremity tendon reflexes were normal. Bilateral Babinski and Chaddock signs were positive. Meningeal stimulation sign was negative.

### 3.3 Auxiliary examinations

Laboratory tests: Routine blood work, liver/renal function, electrolytes, tumor markers, thyroid function, coagulation profile, D-dimer, homocysteine, folic acid, and vitamin B12 were all within normal ranges.

#### 3.3.1 Imaging studies

Cranial CT: Bilateral periventricular and centrum semiovale WM demyelination.

**FIGURE 1:**
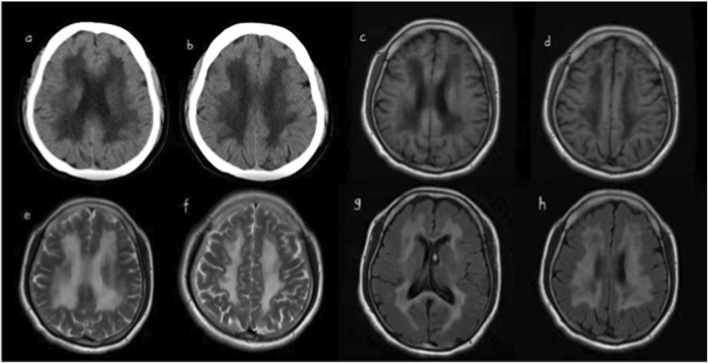
**(a, b)** are cranial CT scans, showing demyelinating changes in the periventricular white matter and white matter of the semioval center bilaterally. Hypointense signals are visible in the anterior and posterior horns of the lateral ventricles on T1-weighted images **(c,d)**. Slightly hyperintense signals are displayed in the anterior and posterior horns of the lateral ventricles on T2-weighted images **(e,f)**. Extensive demyelinating changes in the periventricular white matter and white matter of the semioval center can be observed on T2-FLAIR images **(g,h)**.

Cranial MRI + DWI: Patchy abnormal signals in the bilateral periventricular and centrum semiovale regions (low signal on T1WI, high signal on T2WI). FLAIR sequencing showed hypointense liquefaction signals near the anterior/posterior horns of the lateral ventricles, consistent with CSF-like intensity (Figure 1).

Electrocardiogram、cardiac/abdominal/cerebrovascular/neck color ultrasound、chest CT and craniocerebral CTA revealed no abnormalities.

#### 3.3.2 Genetic testing

**FIGURE 2:**
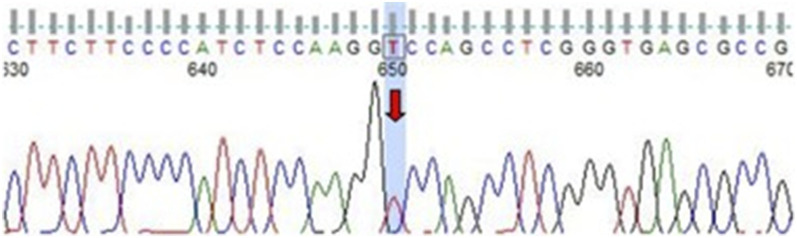
Sanger sequencing result of EIF2B5 c.185A>T (p.Asp62Val).

With ethical approval (General Hospital of Ningxia Medical University) and informed consent, peripheral blood was collected for next-generation sequencing (NGS) of hereditary leukoencephalopathy-related genes. A homozygous missense variant in EIF2B5 was identified: NM_001414.4:c.185A>T (p.Asp62Val) (Figure 2). This variant was absent from the ESP6500siv2_ALL and 1000g2015aug_ALL databases but registered in dbSNP147 (rs1560105986). Despite efforts to obtain parental genetic samples for zygosity validation, we were unable to secure these specimens due to practical and ethical constraints. The patient’s parents reside in a geographically remote rural area with limited access to healthcare facilities, making sample collection logistically challenging and cost-prohibitive for the family. Additionally, after comprehensive informed consent counseling—including detailed explanations of the purpose of parental testing, potential implications for inheritance pattern clarification, and the limitations of zygosity interpretation without their data—the parents declined participation due to personal concerns regarding genetic information disclosure and lack of perceived direct clinical benefit to themselves.

**FIGURE 3:**
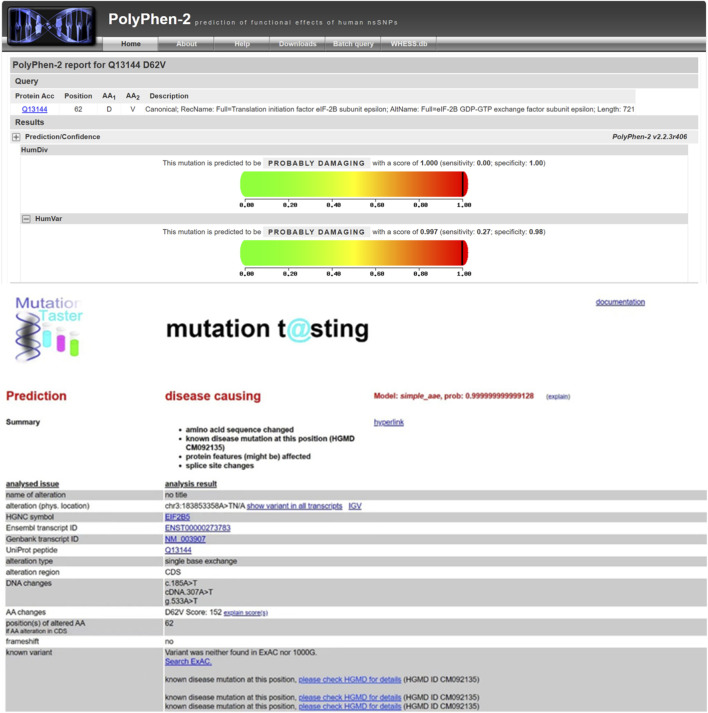
Pathogenicity prediction: Both MutationTaster and PolyPhen-2 predicted that this mutation site may induce disease occurrence.

While Sanger sequencing confirmed the presence of the EIF2B5 c.185A>T variant in the patient’s genome and showed consistent homozygous signal intensity across replicate assays, the absence of parental data introduces inherent limitations. Specifically, we cannot fully exclude the possibility of allelic dropout (ADO) during sequencing or uniparental disomy (UPD)—scenarios that could erroneously mimic a homozygous call despite an underlying heterozygous genotype. To mitigate this uncertainty, we cross-validated the variant with multiple bioinformatics tools (MutationTaster, PolyPhen-2) and referenced prior functional studies confirming its pathogenicity (Figure 3), which collectively support the clinical relevance of the variant. (Leng et al., 2011). Nevertheless, parental genetic testing would have been invaluable for definitive zygosity confirmation and inheritance pattern elucidation, and this limitation is acknowledged in the study’s discussion.

### 3.4 Diagnosis

Combined with clinical manifestations (adult-onset headache, cognitive decline, menstrual irregularities), radiological features (symmetrical WM abnormalities with CSF-like liquefaction), and genetic confirmation (EIF2B5 c.185A>T homozygosity), the final diagnosis was adult-onset VWMD.

## 4 Literature review and discussion

### 4.1 Epidemiology and clinical spectrum of adult-onset VWMD

Our literature review of 99 genetically confirmed adult-onset VWMD cases (Table 1) showed that EIF2B5 is the most frequently mutated subunit (68/99 cases, 68.7%), followed by EIF2B3 (16/99, 16.2%), EIF2B2 (6/99, 6.1%), EIF2B4 (5/99, 5.1%), and EIF2B1 (4/99, 4.0%). Female patients predominated (81/99, 81.8%), likely reflecting the association between VWMD and POF in females (Su et al., 2022). Initial symptoms varied by genotype (Table 1):

**TABLE 1:** Gene, sex, and first symptoms of 99 patients with delayed-onset VWM in China and abroad (Benzoni et al., 2023; Ren et al., 2022; Xu et al., 2021).

Gene	Number of patients	Gender		First symptom						
	(n = 99)/n (%)	M (n = 18)/n	F (n = 81)/n	CA	CD	Of	H	PS	S	Others
EIF2B1	4	2	2	1	1	—	—	—	1	​
EIF2B2	6	1	5	4	1	—	1	—	1	​
EIF2B3	16	0	16	5	3	2	2	2	3	DV
EIF2B4	5	1	4	4	1	—	—	—	—	​
EIF2B5	68	14	54	31	9	7	6	11	3	U

M: male; F: female; CA: cerebellar ataxia; CD: cognitive decline; OF: ovarian failure; H: headache; PS: psychiatric symptom; S: seizures; DV: decreased vision; U: uracratia.

EIF2B5: Cerebellar ataxia (31/68, 45.6%), psychiatric symptoms (11/68, 16.2%), cognitive decline (9/68, 13.2%), ovarian failure (7/68, 10.3%), and headache (6/68, 8.8%) were the most common initial manifestations.

EIF2B3: Cerebellar ataxia (5/16, 31.3%), cognitive decline (3/16, 18.8%), and seizures/psychiatric symptoms (2/16 each, 12.5%) were predominant; 2 cases (12.5%) presented with ovarian failure.

EIF2B1/EIF2B2/EIF2B4: Cerebellar ataxia and cognitive decline were the most frequent initial symptoms; no ovarian failure was reported in EIF2B1 or EIF2B4 cases.

Notably, adult-onset VWMD often presents with atypical symptoms compared to childhood-onset disease. While childhood cases typically manifest with severe ataxia and rapid progression (Abbink et al., 2019), adults more commonly present with milder, progressive cognitive impairment, psychiatric symptoms, or headaches (Damásio, 2012; Ho et al., 2020). Our patient’s initial symptom (intermittent headache) aligns with this adult-specific pattern, highlighting the need for clinical vigilance in patients with chronic headaches and subtle WM abnormalities on MRI.

### 4.2 Genotype–phenotype correlations in EIF2B subunits

Genotype–phenotype correlations in adult-onset VWMD are complex but reveal key trends:

EIF2B5: As the most commonly mutated subunit in adults, EIF2B5 variants are strongly associated with ovarian failure (7/68 adult cases, 10.3%), consistent with previous reports that EIF2B5 mutations are the primary cause of OLD (Su et al., 2022). Our patient had menstrual irregularities and infertility, but lack of gynecological ultrasound and sex hormone testing precluded a POF diagnosis—underscoring the importance of comprehensive evaluation for female adult-onset VWMD patients.

EIF2B3: Female predominance (16/16 cases) and a higher rate of non-motor symptoms (e.g., psychiatric symptoms, vision loss) distinguish EIF2B3-related adult VWMD.

EIF2B1/EIF2B2/EIF2B4: These subunits are less frequently mutated in adults, with a higher proportion of motor symptoms (e.g., ataxia) as initial manifestations. No clear association with ovarian failure was observed, suggesting subunit-specific effects on ovarian function (Hamilton et al., 2018).

Functional differences between EIF2B subunits may explain these correlations. The EIF2Bε subunit (EIF2B5) has guanine nucleotide exchange factor (GEF) activity, which is critical for eIF2B complex function (Abbink et al., 2019). Variants in EIF2B5 may disrupt both neuronal and ovarian tissue homeostasis, whereas variants in other subunits (e.g., EIF2B1–EIF2B4) primarily affect neuronal function. However, no strict genotype–phenotype correlation has been established, likely due to modifiers such as genetic background, environmental stressors, and age-related cellular resilience.

### 4.3 Pathogenic mechanism: Haploinsufficiency vs. biallelic loss of function

Haploinsufficiency (pathogenesis due to one nonfunctional allele) is not a relevant mechanism for VWMD. As an autosomal recessive disorder, VWMD is driven by biallelic loss of function (LOF) of EIF2B subunits (Keefe et al., 2020). Our patient’s homozygous EIF2B5 variant disrupts eIF2B complex activity, triggering sustained ISR activation and WM damage (Keefe et al., 2020). Haploinsufficiency was not considered in this case, as heterozygous EIF2B variants are typically non-pathogenic.

### 4.4 Novelty and contribution of this case

The EIF2B5 c.185A>T (p.Asp62Val) variant was previously reported only in a pediatric patient (Leng et al., 2011), making our case the first documentation in an adult. This expands the variant’s age-related spectrum, demonstrating that pediatric-associated EIF2B variants can manifest in adulthood with milder, atypical symptoms. Key contributions include:

Confirming that c.185A>T is not restricted to childhood-onset VWMD, challenging the assumption that pediatric variants are incompatible with adult presentation.

Highlighting headache as an underrecognized initial symptom of adult-onset EIF2B5-related VWMD, which may delay diagnosis if not linked to WM abnormalities.

Reinforcing the value of genetic testing for adult patients with unexplained cognitive decline, headaches, or menstrual irregularities and suspicious MRI findings.

### 4.5 Diagnosis and management

Definitive VWMD diagnosis requires a combination of clinical manifestations, radiological features, and genetic confirmation (Escobar-Pacheco et al., 2024). MRI findings (diffuse symmetrical WM abnormalities with CSF-like signal intensity) are highly suggestive, but genetic testing is the gold standard (Damásio, 2012).

Currently, no curative treatment for VWMD exists. Management focuses on:

Symptomatic relief (e.g., analgesics for headaches, cognitive rehabilitation).

Avoiding stressors (infection, trauma, emotional distress) that exacerbate ISR activation and disease progression (Deng et al., 2021).

Prenatal genetic counseling for affected families, as early diagnosis reduces familial and societal burdens (Fogli et al., 2003).

Our patient was advised to avoid stress, undergo regular clinical and imaging follow-up, and complete gynecological evaluation for POF. She continues symptomatic treatment with close monitoring.

### 4.6 Study limitations

This study is subject to several limitations that warrant acknowledgment. First, we did not explore gene-gene interactions, which may serve as key modulators of disease severity, phenotypic heterogeneity, and age of onset. As integral components of a sophisticated molecular regulatory network, EIF2B subunits may interact with other genes involved in the integrated stress response (ISR) or myelination processes, and such crosstalk could potentially alter the clinical penetrance of the c.185A>T variant. Second, the unavailability of parental genetic data compromises our capacity to definitively confirm the homozygous status of the identified variant. Although Sanger sequencing verified the presence of the variant in the patient’s genome, it fails to rule out allelic dropout (ADO) or uniparental disomy (UPD)—two scenarios that may lead to misinterpretation of zygosity. Moreover, parental genetic testing would have facilitated the exclusion of *de novo* variants and the clarification of the underlying inheritance pattern. Third, the paucity of long-term follow-up data precludes a comprehensive characterization of the disease progression trajectory associated with this variant in adult patients. To address these research gaps and refine our understanding of EIF2B5-related VWMD, future investigations should enroll larger cohorts, incorporate parental genetic validation, and conduct systematic gene interaction analyses.

## 5 Conclusion

This case report describes the first adult with VWMD harboring the EIF2B5 c.185A>T (p.Asp62Val) variant, expanding the phenotypic and age-related spectrum of this rare disease. A literature review of 99 adult-onset VWMD cases highlights genotype–phenotype correlations: EIF2B5 is the most frequently mutated subunit in adults, strongly associated with ovarian failure in females. Adult-onset VWMD often presents with atypical non-motor symptoms (e.g., headache, cognitive decline), emphasizing the need for clinical vigilance and early genetic testing.

Notably, this study is limited by the lack of gene-gene interaction analyses and parental genetic data for definitive zygosity validation. Despite these limitations, our findings underscore the importance of considering pediatric-associated EIF2B variants in adults with unexplained white matter abnormalities. While no curative treatment exists, early diagnosis enables prenatal counseling, stress avoidance, and symptomatic management to slow progression. Future studies should explore subunit-specific pathogenic mechanisms, gene-gene interactions, and disease severity modifiers to improve patient outcomes.
